# Utility of handgrip strength (HGS) and bioelectrical impedance analysis (BIA) in the diagnosis of sarcopenia in cirrhotic patients

**DOI:** 10.1186/s12876-022-02236-7

**Published:** 2022-03-30

**Authors:** Lalida Luengpradidgun, Naichaya Chamroonkul, Pimsiri Sripongpun, Apichat Kaewdech, Pramot Tanutit, Natee Ina, Teerha Piratvisuth

**Affiliations:** 1 Gastroenterology and Hepatology Unit, Division of Internal Medicine, Faculty of Medicine, Prince of Songkla University, Hat Yai, 90110 Thailand; 2 Division of Radiology, Faculty of Medicine, Prince of Songkla University, Hat Yai, 90110 Thailand; 3 NKC Institute of Gastroenterology and Hepatology, Faculty of Medicine, Prince of Songkla University, Hat Yai, Songkhla, Thailand

**Keywords:** Sarcopenia, Cirrhosis, Handgrip strength, BIA

## Abstract

**Background:**

Sarcopenia is associated with disability, mortality, and poorer survival in cirrhotic patients. For the evaluation of muscle volume, computed tomography (CT) is the most accurate tool. Unfortunately, it would be hard to apply a muscle mass measuring CT to daily practice. This research aims to study the utility of handgrip strength (HGS) and bioelectrical impedance analysis (BIA) to detect sarcopenia in cirrhotic patients compared with CT as the reference.

**Methods:**

In cirrhotic patients who met inclusions criteria (age 20–70 years, ascites < grade 2 of International Ascites Club grading system, no active malignancy, and no cardiac implanted device), HGS were measured using a Jamar dynamometer. Subsequently, patients with low muscle strength (defined as JSH criteria, < 26 kg in male, < 18 kg in female) were then underwent CT and BIA (Tanita MC780 MA) on the same day to measure muscle volume, the definition of sarcopenia by CT was according to the Japan Society of Hepatology (JSH). We also collected data from patients with normal HGS whose CT results were available in the study period.

**Results:**

From 146 cirrhotic patients who underwent HGS, 30 patients (20.5%) had diagnosed low HSG. Data from 50 patients whose available CT results included 30 low HGS and 20 patients with normal HSG. The HGS was strongly correlated with skeleton muscle index (SMI) by CT (r = 0.81, *p* < 0.001) and had an excellent diagnostic performance for detecting sarcopenia by using JSH criteria the sensitivity, specificity, NPV and PPV were 88.2%, 100%, 100%, and 98.7% respectively. In contrast, only 6 of 30 patients (20%) met sarcopenic criteria by BIA. Among sarcopenic patients, the result showed a fair correlation between SMI and BIA (r = 0.54; *p* < 0.002).

**Conclusion:**

Our study demonstrated an excellent correlation between HGS and SMI by CT in the mixed cirrhotic population from the sarcopenia and non-sarcopenia groups. The HGS using the JSH criteria showed an excellent performance in detecting sarcopenia compared to CT. Nonetheless, for the BIA by using the current cut-offs demonstrated unacceptable rate to detect sarcopenia.

**Supplementary Information:**

The online version contains supplementary material available at 10.1186/s12876-022-02236-7.

## Background

Sarcopenia is defined by progressive and generalized skeletal muscle degeneration [1], it can be categorized as primary and secondary sarcopenia. Unlike primary sarcopenia, which is associated with aging, secondary sarcopenia results from chronic conditions such as malignancy, rheumatoid arthritis, cirrhosis, etc. [2, 3]. Sarcopenia affects 20–70% of cirrhotic patients, especially in those with advanced disease [4–6]. This condition predicts higher morbidity and mortality in cirrhotic patients, including those who are liver transplantation recipients [5, 7], increases risks for hepatic encephalopathy, hepatocellular carcinoma [8], and also results in reduced quality of life [9, 10].

Several criteria to define sarcopenia have been proposed, but three standard consensuses were established: European Working Group on Sarcopenia in Older People (EWGSOP) [3], Asian Working Group for Sarcopenia (AWGS) [8] and Japan Society of Hepatology (JSH) [11] guidelines for sarcopenia in liver disease. The AWGS and JSH criteria are considered suitable for Asian patients that have different body compositions from those who are Caucasians.

There are various tools that can be used to demonstrate sarcopenic state, e.g., Bioelectrical impedance analysis (BIA), dual X-ray absorptiometry (DEXA), and CT scan for muscle volume measurement. Indeed, CT imaging for an evaluation of body composition is precise, objective, and currently counted as the gold standard approach to diagnose skeletal muscle abnormalities by measuring the cross−sectional muscle area (cm^2^) at the level of the third lumbar vertebrate (L3), normalized to the patient’s height and reported as skeleton muscle index (SMI). Unfortunately, the precise tool for assessing muscle volume as CT is not practical for routine care, costly, and a radiation-exposed method.

BIA is a commonly used method for estimating body composition, in particular, body fat and muscle mass, using a weak electric current that flows through the body [12]. There were data of BIA, when compared to CT, showed a moderate correlation for muscle mass evaluation in colorectal malignancy patients [13] and also showed a good correlation with a correlation coefficient (r) of 0.72 in patients with chronic liver disease [11]. Therefore, we aim to study the correlation of simple bedside tools as BIA and handgrip strength (HGS) compared with CT for detection of low muscle volume state in cirrhotic patients. The secondary aim is to investigate the prevalence of low SMI among cirrhotic patients who have low HGS.

## Methods

This study was conducted at the Gastroenterology and Hepatology Outpatient Department at our center, which is a tertiary care university hospital in Thailand, between June 2019 and March 2021. The study was approved by the office of the human research ethics committee, Faculty of Medicine, Prince of Songkla University; IRB number 62-349-14-1.

### Patient characteristics

We enrolled patients aged between 20 and 70 years, who met inclusions criteria as follows: (1) Cirrhotic liver (diagnosed either by imaging [ultrasonography or CT or magnetic resonance imaging] or histology proven), (2) Agreed to sign informed consent. The exclusion criteria were: (1) Active hepatocellular carcinoma or other malignancies, (2) Clinically significant ascites detected by physical examination which is comparable to ascites grade ≥ 2 by International Ascites Club grading system, (3) Hepatic encephalopathy ≥ grade 2 by West Haven criteria, (4) Patients with a cardiac pacemaker or implanted medical device, (5) Pregnancy or lactation, and (6) Model for end Stage Liver Disease (MELD) score ≥ 20 points.

HGS was then measured using a Jamar dynamometer in all eligible patients. Following the HGS test, only patients who met the criteria of low HGS according to JSH criteria would undergo BIA and CT according to the study protocol. Additionally, the cirrhotic patients with available CT, including L3 area within 2 weeks before HGS but had normal HGS results, were also included in this study as a comparator group (This group of patients did not undergo BIA testing).

## Assessment of HGS and skeletal muscle mass from BIA and CT scan

### Handgrip strength test (HGS)

HGS was measured in all patients (both dominant and non-dominant hand, maximum squeeze at least 2 s, 3 trials, and average results were used). The strength measurement was performed in 90 degrees elbow flexed position, using a Jamar dynamometer. The HGS of < 26 kg in males and < 18 kg in females is considered as low HGS according to JSH criteria.

### Bioelectrical impedance analysis (BIA)

BIA was measured only in patients who had low HGS on the same day, using a portable BIA device (Tanita MC780 MA, Tokyo, Japan), 8-electrode configuration, and testing time within 30–60 s. Participants were instructed to avoid vigorous exercise at least 24 h before test, to finish the last meal at least 2.5-h before the measurement, to empty their bladder before the measurement and also removed all metallic objects (e.g., jewelry, keys). The skeletal muscle mass was automatically calculated from the device. After each assessment, the results were calculated into kilograms for each limb, then the muscle masses from 4 limbs were summed up, referred to as an appendicular skeletal muscle (ASM). To determine muscle volume, the SMI by BIA was calculated by using formula: ASM (kg)/ht^2^. The SMI cut-offs by BIA for the diagnosis of sarcopenia was based on the JSH criteria: < 7.0 kg/m^2^ in males and < 5.7 kg/m^2^ in females.

### Computed tomography (CT)

Non-contrast CT scan of the abdomen was performed in all low HGS patients to measure the total cross-sectional area (CSA) of the muscles (psoas, erector spinae, quadratus lumborum, transverse abdominal, internal oblique, external oblique, and rectus abdominis) by Toshiba Aquilion prime CT scanner. In order to calculate the skeletal muscle index: SMI (cm^2^/m^2^) area of L3 vertebra level was selected according to the evidence of prior study revealed the strongest associations with total skeleton muscle volume were found for single-slice measurements obtained at L3/4 (r = 0.94) [14]. For skeletal muscle analysis, we adapted semiautomatic software developed by Jae-Hoon Kim et al. [15] which revealed an excellent intrareader reproducibility for assessment of skeletal muscle area [ICC = 0.996; 95% confidence interval (CI), 0.979–0.999; P < 0.001] from prior study [16]. All measurements were performed by an experienced radiological technician and a board-certified radiologist in abdominal diagnostic imaging and body composition analysis who were blinded to the BIA and HGS results. Details of semiautomatic software was described in Additional file 1. The SMI cut-offs by CT for the diagnosis of sarcopenia based on the JSH criteria were < 42 cm^2^/m^2^ in males, and < 38 cm^2^/m^2^ for females. All low HGS patients underwent CT on the same day after BIA test.

### Definition of sarcopenia

Sarcopenia was diagnosed when cirrhotic patients had low muscle strength (male < 26 kg, female < 18 kg) plus low muscle mass (SMI) by CT (male < 42 cm^2^/m^2^, female < 38 cm^2^/m^2^) according to the JSH criteria.

### Statistical analysis

A sample size of at least 30 patients was required to demonstrate the expected correlation coefficient between SMI by BIA and SMI by CT scan of 0.72, with alpha = 0.05 and beta = 0.2 from the sample size calculation. Descriptive statistics were presented as mean ± standard deviations (mean ± SD) or median (interquartile range; IQR) for continuous variables depending on the distribution of the data and as a percentage for categorical variables. The prediction of low SMI state (CT criteria) by HSG was assessed by sensitivity and specificity given the JSH criteria cut-offs. Spearman’s rank correlation test was used to verify the correlation of muscle mass between CT scans and BIA, HGS and BIA, and HGS and CT scans. A *p*-value of < 0.05 was considered to be statistically significant.

## Results

### Sarcopenic and nonsarcopenic patients

A total of 146 patients who visited the Gastroenterology and Hepatology Outpatient Department during the study period were considered eligible. After HGS was performed, there were 30 patients who had low HGS (16.5%). All of these 30 patients underwent both CT scan and BIA, while the rest of 116 patients who had normal HGS results, their medical records were reviewed. Of those, 20 patients had CT scan data in which SMI could be calculated (CT performed within 2 weeks of enrollment date). Finally, there were 50 patients with available CT and HGS data, and only 30 of 50 patients had all CT, HGS and BIA data.

Of 50 patients who had CT scan results, 28 were male and 22 were female. The mean age was 57.8 and 62.3 years for males and females, respectively. The mean BMI was 24.7 and 25.7 kg/m^2^ for males and females, respectively. Chronic HBV infection was the most common of cirrhotic etiology, 28.6 and 40.9% for males and females, respectively. For Child–Pugh classification, 86% and 77% of males and females were in CTP-A. The median HGS results were 28.6 kg in male and 16 kg in female patients. For males and females, the mean SMI by CT was 43 and 31 cm^2^/m^2^, respectively. Having both low HGS and low muscle mass by CT according to JSH criteria were used to define sarcopenia.

The characteristics of cirrhotic patients with and without sarcopenia are shown in Table 1. Among 30 sarcopenic patients, the median age was older, although not statistically significant, than non-sarcopenic patients. The majority of sarcopenic patients were found to be women. The etiology of cirrhosis was not different in both. The mean BMI in sarcopenic patients was significantly lower than those without (23.7 vs 27.2 kg/m^2^, *p* = 0.006). Likewise, serum albumin (3.6 vs 4.2 g/dL, *p* = 0.002) and platelet count (114 vs 158 × 10^9^/L) in sarcopenic patients were significantly lower than those without. As specified by the definition, sarcopenic patients had significantly lower HGS (mean 16.7 vs 31.3 kg) and SMI by CT (mean 32.3 vs 45.3 cm^2^/m^2^) (all *p* < 0.001).

**Table 1:** Clinical and physical characteristics of all patients and according to sarcopenic status

	Total (n = 50)	Without sarcopenia (n = 20)	With sarcopenia (n = 30)	*p*-value*
Age (year) (median, IQR)	63 (54.5, 64.5)	58 (51, 64)	63 (58.2, 66.5)	0.058
Gender (female) (%)	22 (44)	3 (15)	19 (63.3)	0.002
BMI (kg/m^2^) (mean ± SD)	25.1 (4.5)	27.4 (4.8)	23.7 (4.2)	0.006
*Etiology of cirrhosis*				
Alcohol	7 (14)	3 (15)	4 (13.3)	0.451
Viral hepatitis B	17 (34)	10 (50)	7 (23.3)	
Viral hepatitis C	8 (16)	3 (15)	5 (16.7)	
NAFLD	5 (10)	1 (5)	4 (13.3)	
AILD	2 (4)	0 (0)	2 (6.7)	
Other	3 (6)	0 (0)	3 (10)	
> 1 etiology	8 (16)	3 (15)	5 (16.7)	
Diuretic use (%)	6 (12)	1 (5)	5 (16.7)	0.381
History of HCC	11 (22)	6 (30)	5 (16.7)	0.311
*Laboratory variables*				
TB (mg/dl), (median, IQR)	1 (0.5, 1.6)	0.9 (0.5, 1.4)	1 (0.7, 2)	0.321
AST (IU/ml), (median, IQR)	42 (30.5, 68.5)	35 (25.5, 52.5)	53 (35.2, 70.5)	0.07
ALT (IU/ml), (median, IQR)	37 (24.2, 40.8)	36 (24.2, 39)	37 (24.2, 43.2)	0.482
ALB (g/dl), (mean ± SD)	3.9 (0.7)	4.2 (0.6)	3.6 (0.6)	0.002
Hb (g/dl), (mean ± SD)	12.6 (2.5)	13.8 (2.3)	11.7 (2.2)	0.003
Platelet (10^9^/l), (mean ± SD)	131.9 (67.4)	158.7 (64.7)	114.1 (64.2)	0.021
INR (median, IQR)	1.2 (1, 1.3)	1.1 (1, 1.2)	1.2 (1.1, 1.4)	0.068
Creatinine (mg/dl), (mean ± SD)	0.8 (0.2)	0.9 (0.2)	0.8 (0.2)	0.015
Child–Pugh score, (median, IQR)	5 (5, 6)	5 (5, 5)	6 (5, 6)	0.011
*Child–Pugh*				
A/B/C	41 /6 /2	18/2/0	23/5/2	0.505
MELD score, (median, IQR)	9 (7,11.8)	8 (6, 10)	9 (7, 12.8)	0.208
Mean HGS, (median, IQR)	21.7 (16.5,30.1)	31.3 (28.6, 34.2)	16.7 (15, 18.4)	< 0.001
SMI (cm^2^/m^2^), (mean ± SD)	37.5 (8.8)	45.3 (6.7)	32.3 (5.6)	< 0.001

BMI, body mass index; NAFLD, nonalcoholic fatty liver disease; ALID, autoimmune liver diseases; TB, total bilirubin; AST, aspartate aminotransferase; ALT, alanine aminotransferase; ALB, albumin; INR, international normalize ratio; MELD score, model for end stage liver disease; HGS, handgrip strength; SMI, skeletal muscle index

### HGS as a single tool for predicting SMI by CT

**Fig. 1:**
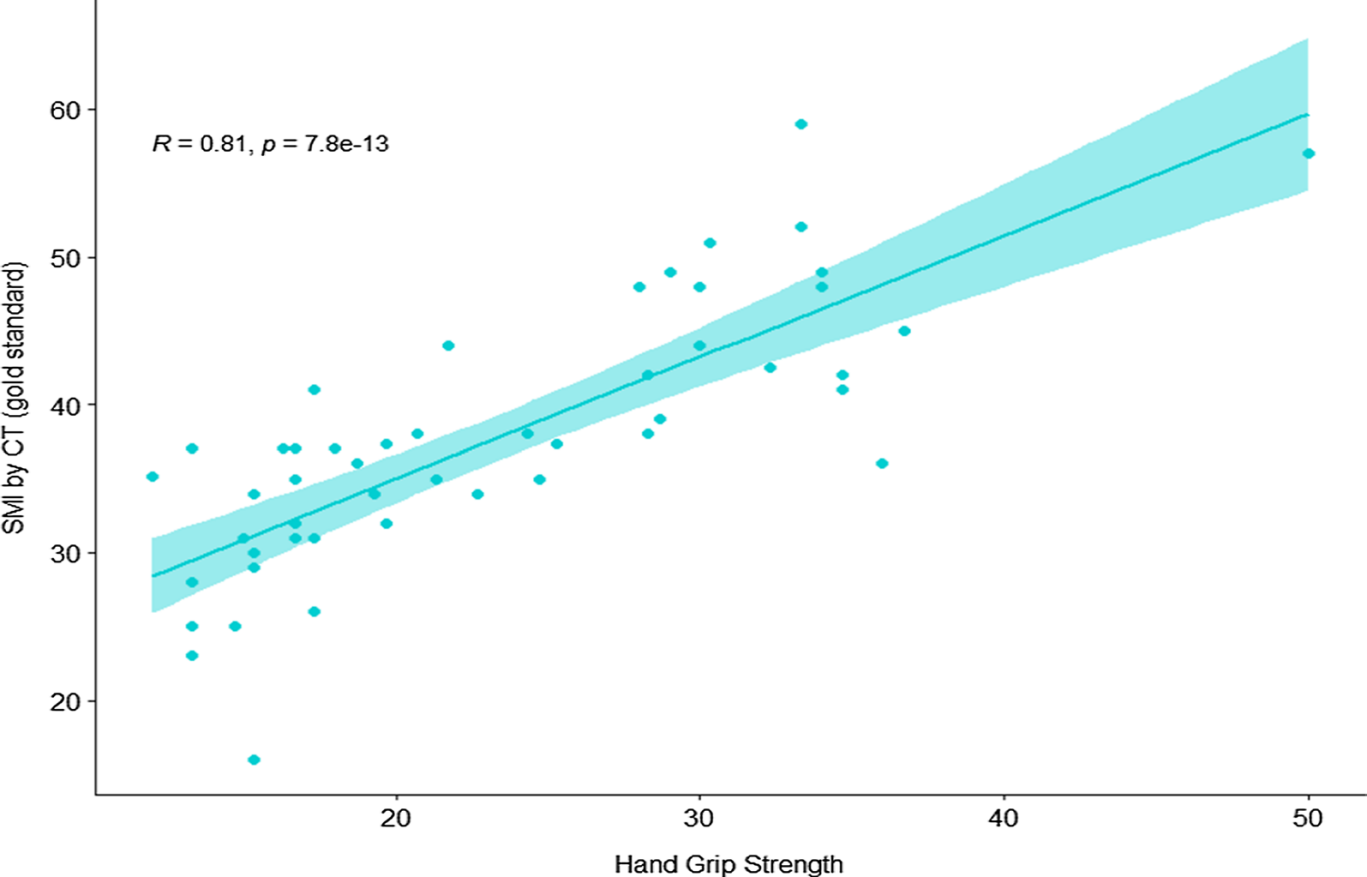
Correlation between HGS and skeletal muscle index by CT (n = 50, all patients with available CT results)

Interestingly, for the entire eligible patients with available HGS and CT results (n = 50), The HGS was strongly correlated with SMI by CT (r = 0.81, *p* < 0.001) (Fig. 1).

Regarding to the sensitivity, specificity, positive predictive value and negative predictive value of HGS for all patients to low SMI by CT according to different HGS cut-offs using JSH and EWGSOP criteria are shown in Table 2.

**Table 2:** The diagnostic performance of the HGS to predict low SMI by CT (n = 50)

HSG cut-offs	Sensitivity (%)	Specificity (%)	NPV (%)	PPV (%)
JSH*	88.2	100	98.7	100
EWGSOP**	94.1	81.2	99.2	82.5

JSH*, Japan Society of Hepatology (HGS < 26 kg for male, < 18 for female); EWGOSP**, European Working Group on Sarcopenia in Older People (HGS < 30 kg for male, < 20 kg for female); NPV, negative predictive value; PPV, positive predictive value. (PPV and NPV were calculated based on 10% of prevalence of sarcopenia)

### Sarcopenia diagnosis and the correlation of SMI between BIA and CT

All 30 patients who had low HGS according to JSH criteria underwent non-contrast CT abdomen and BIA for muscle mass measuring. When using CT-defined low SMI (CT cut-offs; male < 42 cm^2^/m^2^, female < 38 cm^2^/m^2^), 100% of low HGS patients had low SMI by CT and the diagnosis of sarcopenia by JSH criteria was confirmed in all low HGS patients, however, when using BIA cut-offs (male < 7 kg/m^2^, female < 5.7 kg/m^2^), only 20% (n = 6/30) of patients confirmed to have low SMI by CT.

**Fig. 2:**
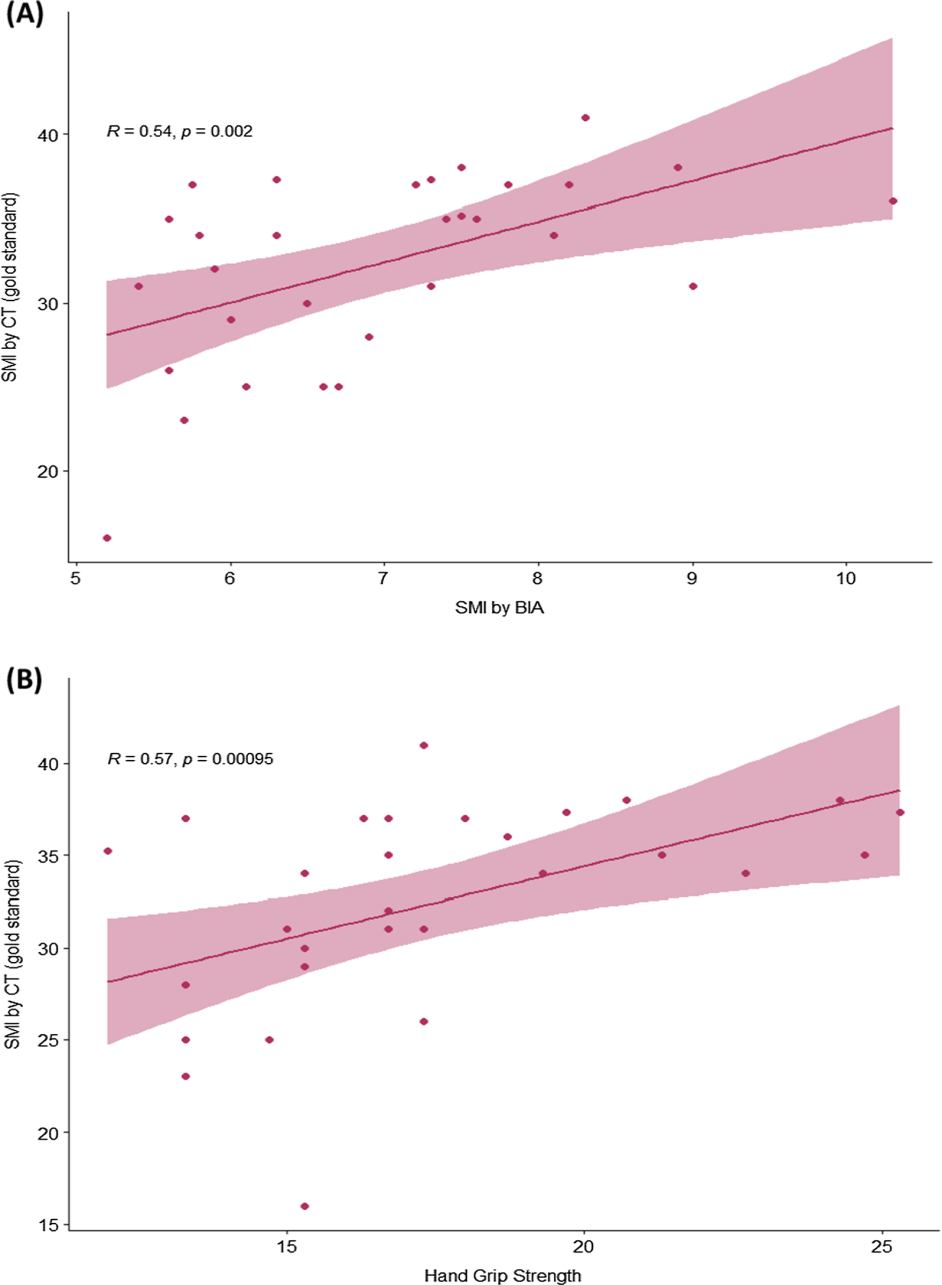
Correlation between skeletal muscle index by CT and **A** BIA, **B** HGS (N = 30, sarcopenia group)

Focusing on the muscle mass (SMI) by CT, we found that in sarcopenic patients, the correlation of SMI by CT and by BIA was fair (r = 0.54, *p* = 0.002) as shown in Fig. 2A. And HGS showed fair correlation with SMI by CT (Fig. 2B) (r = 0.57, *p* = 0.00095).

## Discussion

Sarcopenia is common in cirrhotic patients and associated with several poor outcomes. In our study, among 146 cirrhotic patients for whom HGS were screened, 30 of them (21%) had low HGS; this proportion is similar to previous studies [3, 17]. The criteria for the diagnosis of sarcopenia requires both low muscle strength and low muscle mass; however, CT scan, the standard method to measure muscle mass in the literature, and more specifically, the software to calculate the muscle mass from CT, is not widely available in clinical practice. The BIA method is simple and quick to measure skeletal muscle mass Prior mentioned data from Japan showed a good correlation of SMI measured by BIA with CT (r = 0.72, *p* < 0.01) in 149 chronic liver disease patients [11]. Unlike the study from Japan, our results showed that the BIA was only fairly correlated with CT (r = 0.54, *p* = 0.002). Moreover, when using the recommended BIA cut-offs, only 6 of 30 sarcopenic patients were identified. The performance of BIA to diagnose sarcopenia in our study is lower than expected. The BIA tests are based on the principle of opposition from body tissues to a low intensity alternate electrical current. It estimates fat mass, fat-free mass, skeletal muscle mass, and total body water, using prediction equations based on healthy reference populations. This mechanism leads to hypothesis that BIA might not predict sarcopenia in unhealthy people especially when patients were in the state of fluid retention even, we excluded patients who had more than grade 2 ascites in this study. Nonetheless, the features promote an error or discordance between BIA and CT as reported in the previous study in elderly such as age over 65 years, and BMI < 25 kg/m^2^) [18] were not found in our patients; therefore, other influent factors are considered to be explored in future studies. Another hypothesis is the recent BIA cut-offs that we applied according to JSH criteria might not be appropriated for Thai cirrhotic patients. The results demonstrated BIA and HGS both had a moderate correlation with SMI by CT in patients whose muscle function were low. However, by using the definite cut-offs, HGS still had a very good diagnostic performance to detect evidence of low muscle volume. In contrast BIA could detect only 20% of sarcopenic cirrhosis. Unfortunately, the investigator did not collect the BIA data of the control patients, so the new BIA cut-offs could not be suggested based on data of this study.

HGS was a noninvasive and simple method to evaluate muscle strength. Our data illustrated that using HGS as a single tool for screening low muscle mass was very effective; HGS showed a strong correlation with SMI by CT (r = 0.81, *p* < 0.001). When referring to the accuracy, HGS also had a high sensitivity and specificity for predicting low muscle mass by CT (88.2% sensitivity, 100% specificity). From the results of this study, HGS had an excellent specificity to diagnose low muscle mass by CT, as all patients with low HGS have truly low SMI from CT. Therefore, it might be considered to use as a single test for detecting sarcopenia in Thai cirrhotic patients. Suppose the patients had low HGS (according to JSH criteria). In that case, we might be able to omit CT in order to confirm whether the patients had low SMI or not (the PPV of HGS was 100%), and the treatment intervention can be initiated straightaway. This strategy might aid in saving costs for both patients and payers in terms of detecting sarcopenia status. Nonetheless, if we would like to detect more patients with low skeletal muscle mass (using HGS as a screening tool), the EWGSOP criteria might be more suitable to use as it has a greater sensitivity to diagnose low SMI by CT.

We acknowledge the limitations of our study. First, our study recruited a small sample size which resulted in a restricted power to predict the accuracy of BIA and CT. Second, our study used the Tanita MC 780 BIA machine, which is a small model of the BIA machines aiming for the bedside test; this version might not precisely measure the muscle mass. The different BIA machine models may provide different results. Lastly, we selected ascites grade ≥ 2 by International Ascites Club grading system as an exclusion criterion of patients who have a significance fluid retention which might interference to BIA measurement. The study results might be difference if we expand or narrow this criterion to patients with different stage of ascites.

To concluded, in cirrhotic patients without clinically significant ascites, the HGS test was a simple, inexpensive bedside tool to detect sarcopenic stage and low muscle mass due to the high sensitivity, specificity, and a remarkable correlation with SMI by CT. Even though the BIA (Tanita MC 780) also had a fair correlation with SMI measuring by CT, it underestimated the sarcopenic status when compared with CT when we applied the recent recommended cut-offs.

## Supplementary Information

**Additional file 1**. Details of semiautomatic software.

## Data Availability

Due to ethical restrictions, the dataset related to the current study are available upon request to the corresponding author.

## Abbreviations

AWGS: Asian Working Group for Sarcopenia
BIA: Bioelectrical impedance analysis
CT: Computed tomography
EWGOSP: European Working Group on Sarcopenia in Older People
HGS: Handgrip strength
JSH: Japan Society of Hepatology
SMI: Skeletal muscle index
