# Supplementary material for: Utility of handgrip strength (HGS) and bioelectrical impedance analysis (BIA) in the diagnosis of sarcopenia in cirrhotic patients
Source: BMC Gastroenterol. 2022 Mar 30;22:159. doi: 10.1186/s12876-022-02236-7 (PMC8969388; doi:10.1186/s12876-022-02236-7)
Supplement: Supplementary file 1 — Additional file 1. Details of semiautomatic software. [file 12876_2022_2236_MOESM1_ESM.docx]

**Semiautomatic software for muscle mass measurement protocol**

In this study, we identify muscle volume in CT images for body composition analysis by adaptation of the prior written protocol by Kim SS, et al. ^1^ The open-source software environment (BMI_CT) is available on the following URL (https://sourceforge. net/projects/muscle-fat-area-measurement/). The applications still use the semi-automatic program based on the region-growing segmentation method by increasing regions. The protocol is composed of 3 image processing steps: a thresholding step, a seeding step, and a calculation step. In the thresholding step, the background image (including the CT table and noise) was removed from the original CT image using the Hounsfield unit (HU) (Fig. A). The intensity of preprocessed CT image was linearly transformed into -50 to +150 HU (Fig. B). In the seeding step, muscle areas were identified by pointing at pixels in a lumbar muscle. Then, a signal intensity detection, or Hounsfield value, was saved. The original pixel, known as a seed, was extended by adding contiguous pixels that meet the homogeneity criterion, also known as the threshold range (Fig. C). The trained radiation technologist with over ten years of experience, on the other hand, manually rechecked and filled some areas that leaked (parts of the kidney and bow that gave the same signal) and/or lacked (inadequate area of muscle). The muscle mass area was automatically calculated by the MATLAB program (Fig D). The data demonstrated a significant correlation between this parameter and a clinical outcome in cirrhosis was demonstrated in another cohort and was in the process of submission.


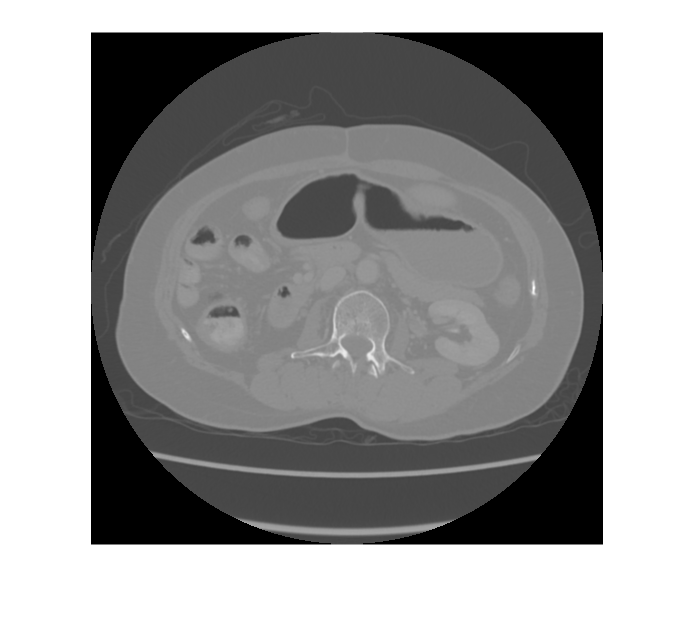

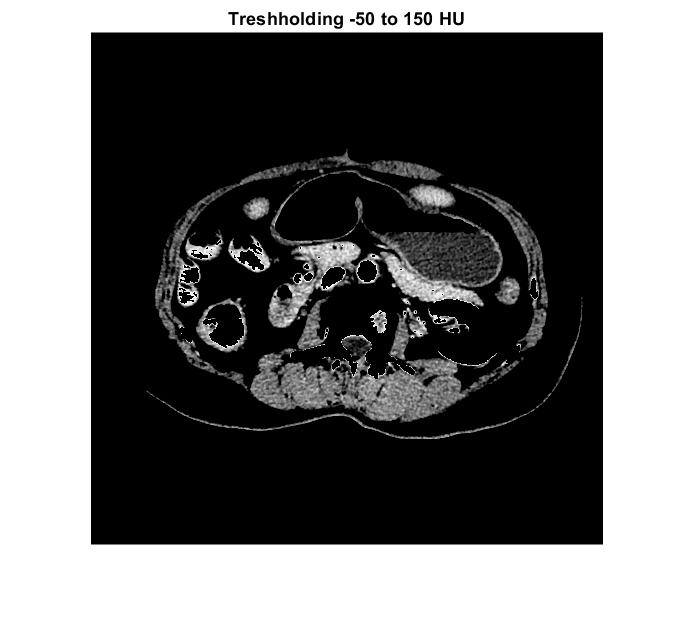


**Fig B:** Thresholding step

Fig B: Thresholding step

**Fig A:** Preprocessing step

Fig A: preprocessing step


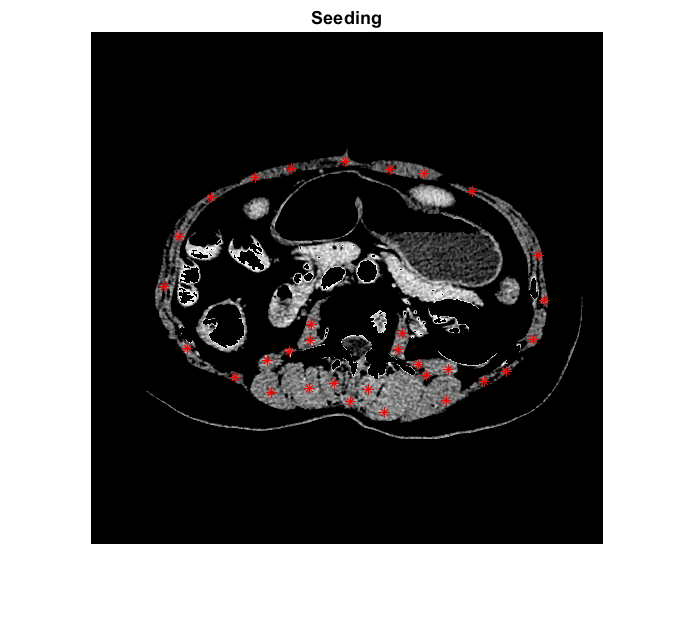

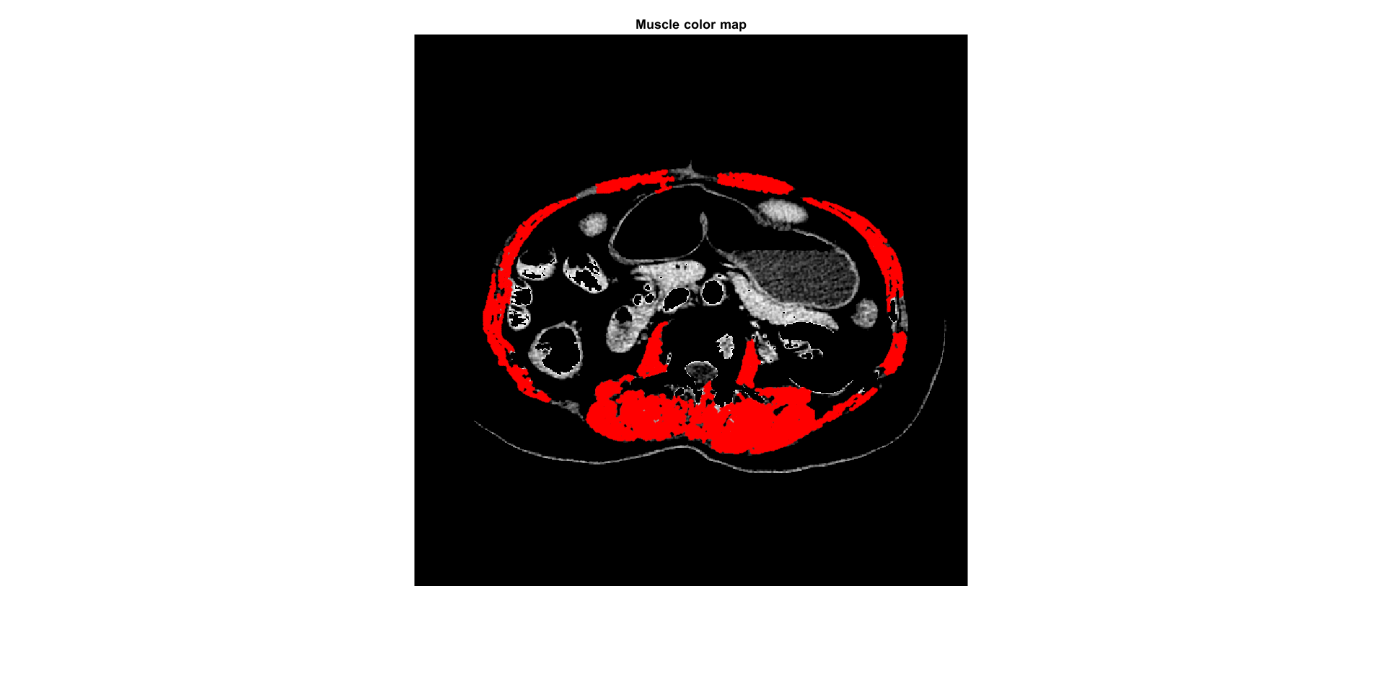


**Fig D:** Muscle color map

**Fig C:** Seeding step

Reference

1. Kim SS, Kim JH, Jeong WK, et al. Semiautomatic software for measurement of abdominal muscle and adipose areas using computed tomography: A STROBE-compliant article. *Medicine (Baltimore)*. May 2019;98(22):e15867. doi:10.1097/md.0000000000015867
